# A Circular Economy Approach to Food Security and Poverty: a Case Study in Food Rescue in Sri Lanka

**DOI:** 10.1007/s43615-023-00255-4

**Published:** 2023-02-17

**Authors:** Nimeshika Aloysius, Jayanath Ananda

**Affiliations:** School of Business and Law, Central Queensland University, Victoria Melbourne, Australia

**Keywords:** Food rescue, Surplus food redistribution, Food waste, Food security, Circular economy

## Abstract

Food rescue has been identified as a sustainable approach in preventing wastage of surplus food and achieving food security. Although food insecurity is widely prevalent in developing countries, there is a paucity of research investigating food donations and rescue operations in these countries. This study focuses on surplus food redistribution activities from a developing country perspective. Specifically, the study analyses the structure, motivations, and limitations of the existing food rescue system in Colombo, Sri Lanka, by conducting a series of structured interviews with twenty food donors and redistributors. The food rescue system in Sri Lanka characterises a sporadic redistribution, and food donors and rescuers are mainly driven by humanitarian motives. The findings also reveal missing institutions — facilitator organisations and back-line organisations — in the surplus food rescue system. Food redistributors identified that inadequate food logistics and establishing formal partnerships as major challenges in food rescue operations. Establishing intermediary organisations such as food banks to provide the required food logistics, imposing food safety parameters and minimum quality standards required for surplus food redistribution, and community awareness programmes on food redistribution can increase the efficiency and effectiveness of food rescue operations. There is an urgent need to embed food rescue as a strategy to reduce food wastage and to enhance food security in existing policies.

## Introduction

Food insecurity^1^ is a significant global problem affecting food availability, access, and distribution [1]. According to the Food and Agriculture Organisation, around 12% of the total population in the world was severely food insecure in 2020 and there are 768 million people on average, who suffer from hunger, which is nearly 9.9% of the global population [2]. The COVID-19 pandemic has exacerbated the problem and increased the proportion of people suffer from hunger in 2020 [2]. More than half of the world’s undernourished people are reported in Asia (418 million) which is around 9% of the total population in the region [2]. According to United Nations Environment Programme [3], 12.9% of the population in developing regions suffer from hunger. The same report states that poor people in developing countries spend 50–80% of their income on food, exposing them to price volatility and food insecurity [3].

The issues of food loss, food waste, and food insecurity are specifically reflected in the 2030 agenda for Sustainable Development with the targets of the Sustainable Development Goals (SDGs). The issue of food insecurity is addressed in SDG 2, having a target in ensuring access to safe, nutritious, and sufficient food throughout the year by all people, including infants by 2030. Also, the SDG 2 have targets to end all forms of malnutrition by 2030, to alleviate stunting and wasting in children under 5 years of age, and to address the nutritional needs of adolescent girls, pregnant and lactating women, and older persons by 2025. The SDG 12.3 calls for halving the global food waste at the retail and consumer levels and the reduction of food losses along production and supply chains, including post-harvest losses by 2030 [4].

Nearly one-third of food produced is wasted each year all around the world [5]. Food waste occurs when any edible material that is intended for human consumption is discarded because of consumers’ purchasing decisions or retailers’ and food service providers’ decisions which affect consumer behaviour [6]. This wasted food has significant impacts on the environment through the use of resources such as land, water, energy, and fuel used in producing, manufacturing, packaging, distributing, and preparing food [7]. Therefore, food waste has significant economic, environmental and social consequences which affect the sustainable development of a nation [8].

Global sustainability challenges such as the growing population and the scarcity of resources drive nations towards a circular economy [9]. Circular economy is an approach which strives to avoid waste and to sustain the value of resources [9]. It is a sustainable economic system where materials and energy are reused, recycled, or refurbished and make waste of one stage of the supply chain a resource to another entity [10, 11]. When the principles of circular economy: reduce, reuse, and recycle are applied to food systems, it helps in reducing food waste and enables alleviating hunger [9, 12, 13]. Rescuing surplus edible food and distributing it to hunger is identified as an approach to reuse food [14] and is conceived as a humane practice which reduces social and environmental impacts of food waste [15]. Redistribution of surplus food minimises the food waste from a social perspective [16]. Therefore, food rescue enables circular economy by recovering food waste and sharing among needy consumers encompassing multiple SDGs.

Food rescue has been identified as an approach in preventing wastage of surplus food, alleviating hunger, and achieving food security [17–21]. It creates a “win–win” situation by reducing food insecurity and fighting food wastage [22]. There is a significant increase in demand for food initiatives such as food banks^2^ in both developing and developed countries due to food affordability problems and income losses following COVID-19 pandemic [2]. Though experiencing food insecurity, food waste is a significant problem in Sri Lanka [23]. The Western Province, where the capital city, Colombo is located, generates the highest amount of solid waste in Sri Lanka (33%) which accounts for the highest proportion of food waste in the country [24, 25]. According to the estimates in the year 2017, around 353 tonnes of food waste has been accumulated in the Colombo municipal council area which is roughly half of the total solid waste in the area [26]. Households were identified as the main food waste generator, while the category “food services” is the second largest contributor to food waste (110 tonnes/day) followed by markets and meat shops [26]. Despite the enormity of the issue, there is a dearth of research on food rescue operations in developing countries [22, 27]. Furthermore, these countries have given less attention to contributions of circular economy [28], and thus, this paper should help to reduce the research gap by focusing on food rescue system in Sri Lanka.

### Research Aims

An in-depth understanding of surplus food rescue systems is needed to enhance contributions to food security and circular economy goals. Therefore, the aim of the present study is to analyse the institutional structure, motivations, and constraints of surplus food donors and redistributors in Colombo, Sri Lanka. The main research questions addressed in the study are the following:

1. What are the institutional arrangements of the existing food rescue system?
2. How do the main stakeholder groups organise food rescue activities (surplus food donors and surplus food redistributors)?
3. What are the motivations and constraints of food rescue activities?

The study makes a distinctive contribution to the literature by providing useful insights on the operation of food rescue system from a developing country perspective, which will be helpful in formulating policies to elevate food insecurity and to reduce food waste. In particular, it highlights the missing links in the food rescue system and argues for the establishment of facilitating agencies to strengthen the system. The findings are also useful in achieving Sustainable Development Goal (SDG) 2 of alleviating hunger and the SDG 12.3 of halving the food waste at the retail and consumer levels by 2030 in developing countries.

The remainder of this article is structured as follows. “Food Rescue and Food Redistribution” presents a literature review on food rescue and food redistribution operations and develops a conceptual model of food rescue. In “Methods and Data,” the case study details and the methodology are presented. Findings are discussed in the “Results and Discussion” under six themes: overview of food insecurity and food waste in Sri Lanka, surplus food donation, surplus food redistribution, overview of Colombo’s food rescue system, motives for surplus food donation and redistribution, and challenges in food rescue system. The concluding section provides several policy implications arising from the findings.

## Food Rescue and Food Redistribution

**Fig. 1:**
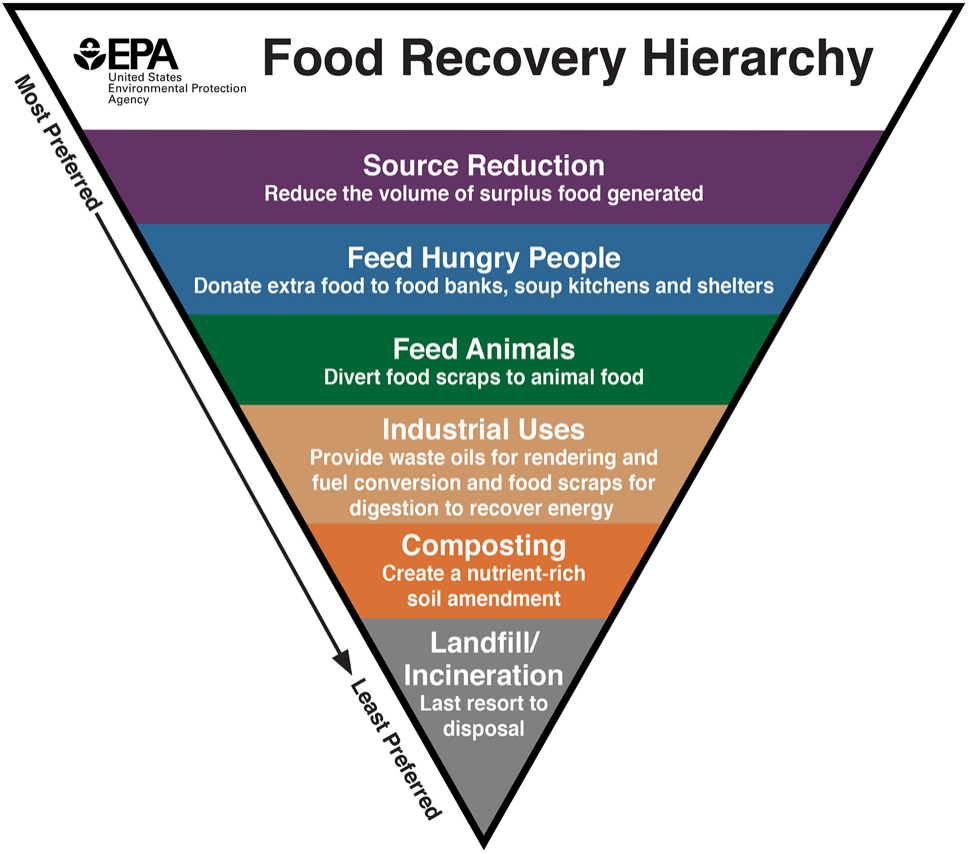
Food recovery hierarchy. Source: US Environmental Protection Agency, https://www.epa.gov/sustainable-management-food/food-recovery-hierarchy

Food rescue, also called food recovery [29, 30] or food redistribution [31, 32], has been identified as one of the key means to address food insecurity and also to prevent wastage of surplus food [17–19, 21]. In the US Food Recovery Hierarchy (Fig. 1) which prioritises various endpoints of surplus food, food rescue is the second most preferred food recovery method. Food redistribution is the practice of collecting surplus edible food and distributing it directly or indirectly to food insecure people [33]. Food redistribution not only divert food waste from landfills but also addresses food poverty [34]. Redistribution of surplus food to the impoverished has been identified as a low-cost approach to reduce the gap between food loss and food insecurity [35, 36]. Food waste prevention through food redistribution avoids negative environmental consequences [37] and also leads to potential social and economic benefits [19, 33]. Vlaholias et al. [31, p. 7998] suggests that food redistribution hold both “pro-social and pro-environmental” aims.

### Food Rescue Operations

There are different modes of food rescue operations in terms of the logistic link between the surplus food owners and the final consumer [38, 39]. These food rescue operations also differ in terms of type of donations accepted, price charged [19], and the final consumer targeted [38]. Food businesses donate surplus food at different stages of the food supply chain, i.e. food production, processing, and retail. These donor organisations include hotels, restaurants, cafes, catering, and supermarkets [18]. Moreover, there are private donors, who donate surplus food from events and special occasions [40]. Surplus food is “most often donated on an ad-hoc basis” [41, p.2]. Motives for donating surplus food includes concern for the environment, concern for people in need, or to decrease landfill costs [31]. Some food businesses donate surplus food as corporate social responsibility (CSR) or as a marketing strategy to win the public favour [31].

**Fig. 2:**
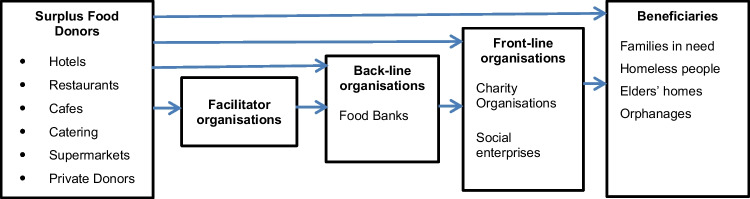
Flow of surplus food redistribution

Organisations that are involved in the redistribution of surplus food can be classified as either “front-line” or “back-line” organisations [40]. Back-line organisations are logistic service providers who transport, store, and redistribute donated food to charitable organisations [41]. A food bank is such an organisation where people donate surplus food, which is stored and further transferred either to a charity or directly to people in need [20, 27]. Although food bank is an established concept in USA and many European countries, it is a recent initiative in many Asian countries [27]. Front-line organisations are charitable organisations which either receive donated food from back-line organisations or collect surplus food directly from donors in the food supply chain and provide this food to their beneficiaries [20, 41]. Back-line organisations must register their suppliers (donors) and the recipients while the front-line organisations only need to record the donors [40]. Figure 2 presents the potential links these organisations have with surplus food donors and beneficiaries.

*Annakshetra* is an initiative in India which rescue excess food from restaurants, temples, weddings, and parties [42]. The initiative strives to reduce food waste and to ensure poverty eradication and food insecurity in India [42]. The 24-h hotline number is circulated among general public in Jaipur area through print media to receive information on surplus food and have a smartphone *App* to connect with surplus food donors [42]. There are around 187 such food redistributing organisations in India; however, they are operating independently, and there is lack of coordination among each other [27]. Most of the food rescue initiatives in India are therefore front-line organisations, where they collect food donations and distribute to needy people immediately. Linking with organisations, which provide logistics for food storage and transport, will allow these initiatives to rescue food and cater food demand irrespective of the geographical areas they operate.

There are various forms of food redistribution operations practiced by surplus food redistributing organisations. Surplus food or food ingredients can either be packed in boxes, parcels, or bags and distributed free of charge to needy people who are unable to purchase them, or can be served in soup kitchens, a place where free food is served to homeless [18, 19, 40]. Sometimes the rescued food is sold at discounted prices [18] by front-line organisations such as social enterprises [43]. Moreover, there are facilitator organisations that play an intermediary role and coordinate between surplus food donors and back-line and/or front-line organisations. These facilitator organisations facilitate food redistribution by matching the supply of surplus food with potential demand [20, 40]. Web platforms and smart phone applications can facilitate food sharing and redistribution by developing a communication channel between food donors and redistributors [14, 39].

Effective utilisation of the recovered food with minimum wastage determines the efficiency of these food rescue operations [20]. While food redistribution operations in developed countries are mediated by facilitator organisations and back-line organisations like food banks [14, 40, 41], this is less prominent in developing countries, which creates inefficiencies in surplus food redistribution activities [27].

The conceptual framework constructed for the research based on the literature review (Fig. 2) represents stakeholders on the supply side (surplus food donors), stakeholders on the demand side (back-line and front-line organisation), and intermediaries (facilitator organisations) involve in surplus food redistribution.

## Methods and Data

### Location of the Study

Colombo city is situated in the Western Province of Sri Lanka and is the commercial capital and the economic centre of the country. Colombo city covers an extent of 37.3 km^2^ and has a resident population of 626,000 [44]. In addition, the daily floating population is nearly 500,000 [44]. Nearly 50% of the population in Colombo city is living in slums with minimum facilities and inadequate living conditions, and urban poverty is most predominant in the city of Colombo [45, 46]. Majority of the urban slum residents in Colombo municipal area are food insecure [45]. Colombo shows the highest percentage (56.2%) of food insecure people, where 59% of households are suffering from food insecurity [26, 47]. People in the Colombo district have significant nutrition related health issues where 40% of them are prevalence to anaemia [26].

### Recruitment of Interviewees

The study adopted a qualitative approach and used structured in-depth interviews to gather information on the surplus food rescue system in Colombo, Sri Lanka. Purposive, non-random sampling technique was adopted to select the interviewees. Data collection was started by identifying organisations involved in surplus food redistribution in Sri Lanka. Two front-line organisations (Robin Hood Army and the Soup Bowl) were selected by referring secondary sources of information, such as published articles, project reports, newspaper articles, social media posts, and websites. Both the above organisations are not-for-profit organisations. A snowball sampling strategy was followed for surplus food donor selection, ensuring the sample represents food producers and retailers. Participants who were actively engaged in surplus food donation and redistribution and who are knowledgeable on managing surplus food in their representative organisations were selected for the interviews. The information on target organisations, stakeholder type (donor or redistributor), and details about the interviewees are presented in Appendix Table 1.

### Data Collection and Analysis

Twenty semi-structured interviews were conducted from February 2021 to July 2021 to collect primary data. The study used semi-structured interview guides to conduct interviews. The interview guides were slightly modified based on the type of the stakeholder, surplus food donor, or redistributor. The interview guide for surplus food donors covered four topic areas: motives for donating surplus food, mode of donation, receivers/beneficiaries, and challenges. The interview guide for surplus food redistributors covered four topic areas: initiation and evolution, motives for food redistribution, the process of food redistribution (suppliers, operations, and communication), and challenges.

Most of the interviews with surplus food donors were conducted face-to-face at the selected organisations. However, the interviews with managers of two hotels were conducted over the phone due to access restrictions imposed as a result of COVID-19 pandemic. Moreover, the interviews with six interviewees representing two food redistribution organisations were conducted over the phone. Each interview lasted between 10 to 20 min. All the interviews were conducted in Sinhala, transcribed manually, and later translated to English. Collected data were analysed by thematic analysis. Transcripts were read multiple times, and quotes in each interview were summarised. Portions of quotes with data relevant to the topics of interest were coded while making comparisons between the two subsamples (surplus food donors and redistributors). Then, the consistencies and patterns in data were screened and similar codes were grouped. Main themes were identified and matched with the literature.

## Results and Discussion

An overview of food insecurity and the magnitude of food waste problem in Sri Lanka are discussed in “Food Waste and Food Insecurity in Sri Lanka.” Findings on surplus food donations in Colombo, Sri Lanka, are discussed in “Surplus Food Donations” followed by two case studies of food redistribution activities in “Surplus Food Redistribution.” The institutional structure of Colombo’s food rescue system is discussed in “Institutional Structure of Colombo’s Food Recue System.” Motives for surplus food donation and redistribution are discussed in “Motives of Surplus Food Donation and Redistribution.” “Food Rescue and Redistribution Challenges” discuss the challenges on food rescue operations and food redistribution activities in Colombo, Sri Lanka.

### Food Waste and Food Insecurity in Sri Lanka

Food waste is a growing problem in Sri Lanka. About 3963 tonnes of food is wasted in the country per day [25, 48]. Currently, more than half of the total solid waste in the country (57%) is food waste [25]. Urbanisation, higher urban income, expansion of supermarket chains, and changes in diets and lifestyles of people are the leading causes for waste in the Sri Lankan food systems [23]. Food waste creates a significant pressure on municipal waste management and negative impacts on the environment mainly in the urban areas of the country, as there are less opportunities to reintegrate these organic waste in the agricultural production [25].

Although a high amount of food is wasted in Sri Lanka, around one-third of the people cannot afford a nutritious diet and approximately 22% of the total population in the country does not have sufficient food to sustain a healthy life [23]. Particularly, urban populations in Sri Lanka have experienced nutritional insecurity [49]. Individuals whose per capita daily calorie consumption is below 2030 kcal are considered as food insecure, according to the official poverty line [47, 50]. Around 17% of children between 6 and 59 months suffer from chronic malnutrition [51]. Nearly 6.8% of Sri Lanka’s population is undernourished, and 15.1% children under 5 years are prevalence to wasting, while 17.3% of them are prevalence to stunting [52]. The highest percentage of food insecure people in Sri Lanka has been reported from the Western Province [53]. The poverty rate in Sri Lanka expected to increase to 11.7% in 2022, due to country’s economic crisis [54], and this will increase the number of food insecure people in the country.

There are more than fifteen acts, ordinances, policies, and by-laws in Sri Lanka addressing different stages of waste management namely, waste collection, segregation, recycling, disposal, waste prevention, and waste reduction. Detailed information on these policies and legislations are presented in Aheeyar [48]. The National Policy on Sustainable Consumption and Production has adopted SDG 12.3 targets, and reducing food waste is a priority area in the National Waste Management Policy. A considerable portion of food which is wasted in Sri Lanka can be re-purposed before expiry for the benefit of the food insecure communities [23]. In 2021, the Ministry of Environment adopted a National Roadmap on Urban Food Waste Prevention and Reduction which was formulated by FAO with the support of International Water Management Institute (IWMI). This roadmap identified the legislative acts and regulations that could be revised to support food redistribution related efforts. This is a promising development as it can draw a significant attention to food rescue operations in Sri Lanka. However, none of the currently active policies and legislations in Sri Lanka has included food rescue operations in their policy statements and goals, as a strategy to manage food wastage or to elevate food security. Moreover, the hygiene requirements, food safety parameters, and minimum quality standards required for leftover food redistribution to needy people have not been included in any of the legal statements currently operative in Sri Lanka [48].

### Surplus Food Donations

Several restaurants, canteens, bakeries, caterers, and supermarkets located in the Colombo city area donate their surplus or unsold food to needy people living in the vicinity [23]. Few surplus food donors in Colombo who strive to limit food waste have collaborated with front-line organisations which are local charities to redistribute the food in good conditions to the families in need [23, 48]. Robin Hood Army is a well-known not-for-profit food redistribution initiative (a front-line organisation) operating in Colombo [23]. The Soup Bowl, another social initiative (a front-line organisation), collects unsold food products from a supermarket chain and directly redistribute them to needy homes [23, 55].

The strategies adopted by surplus food donors in managing surplus food vary between organisations, depending on the type of food and outlet. Some of the strategies expressed were distributing surplus food among employees, donating to homeless, re-using them to produce some other products or as feed, returning the surplus to the main branch, and disposing them systematically as food waste. All the seven interviewees from restaurants and cafes stated that they often distribute surplus cooked food to their employees at the end of the day.

If there is any food remains in the showcases that cannot be sold the following day and excess food in the buffet containers, we allow our waiters and kitchen staff members to take them home at no cost (Manager, Restaurant 1).

It should be noted, however, not every restaurant in Sri Lanka practices this and some restaurants in Sri Lanka have a policy that does not allow giving surplus food to staff. With surplus food give aways to staff, there can be a possibility that staff may have an incentive that might affect sales. For example, this could encourage staff to refill buffets slowly in order to save more food for themselves.

**Fig. 3:**
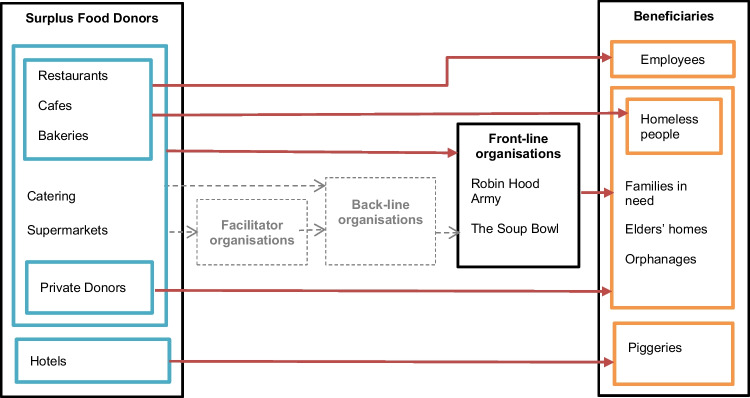
Flow of surplus food redistribution in Colombo, Sri Lanka

Restaurant 2 claimed that they distribute surplus food from their lunch buffet among needy people in nearby area. This is an example for a direct donation to food insecure (Fig. 3).

Each day around 4.00 pm we clean the lunch buffet to prepare tables for our dinner service. If there is excess food from lunch, one of our waiters distribute them to beggars found nearby. As we are doing this for months, some beggars have used to wander near the restaurant close to that time, expecting for a packet of lunch. (Manager, Restaurant 2).

Managers of café 3 and café 4 stated that they donate surplus food to a local charity organisation, which is a front-line organisation. However, they were reluctant to provide information on quantities of food donated. These cafes do not have formal agreements or any written contracts with the charity but used to contact them directly whenever they detect excess food in above the limits they can manage internally.

There are some occasions [that] the café ends up with surplus bakery products, where the quantities are more than that can be shared among our employees. In such situations, I call the RHA (Robin Hood Army) and inform that we have ‘x’ amount of ‘y’ products in excess. They come and collect the surplus in bulk. We have not signed any agreements with RHA, but I personally trust their operations are safe and meet the standards (Manager, Café 3).

Sundgren [41; p. 6] identified this process as “sporadic redistribution” which occurs with “no agreed pick-up schedule among the company and the food aid organisation.” This appears to be the case of food re-distribution in Colombo, Sri Lanka. The donors contact the food redistribution organisations when they detect surplus food, or the food redistribution organisation may contact potential donors. Such a relationship is less predictable than “ongoing redistribution” which often involve the signing of a formal agreement [43].

Sharing and donating surplus food were identified as important and beneficial strategies by the interviewees of restaurants and cafes, because they feed the food insecure people, avoid the guilty feelings on disposing edible food, and reduce the amount of food waste collected.

[Donating surplus food] can help in feeding poor, hungry people around the city and also it avoids the guilty feelings on disposing edible food (Manager, Café 4).

However, the above strategies were not supported by hotels as the below quotes indicate:

There are strict regulations for our staff members on handling food prepared at our main kitchen. We do not allow this food to be shared with anyone outside even though they are surplus and still edible. (Manager, Hotel 1).

We are very keen about the food safety, so we have a policy that we do not allow our guests or staff to bring outside food to the hotel as well as food from the hotel outside. This is mainly because if something happens to a person, like food poisoning, by eating food not under our control, this will harm our reputation. (Manager, Hotel 2).

Two of the large hotels which were interviewed claimed that they send the excess food for livestock feed. These findings are echoed by Jayathilake et al. [25] and Reitemeier et al. [23]. Interestingly, these hotels have partnerships with livestock farms, especially with piggeries, where these farmers and some intermediaries come and collect surplus food in a roster.

We store excess cooked meals in large baskets in a room set with low temperature, where few people come and collect them on every other day. They carry them to piggeries and with this, for sure, both parties are benefitted. It’s good that we do not have to spend money in treating food waste (Manager, Hotel 1).

One of the interviewed hotels had its own biogas generation unit which uses a portion of surplus food. The biogas generated is used to operate a cooking stove in their main kitchen. The usage of renewable energy in hotel operations is one of the environmentally friendly strategies proposed by the management which is consistent with circular economy and sustainability concepts.

Thrice a week, a livestock farm bound with an agreement is collecting the excess food from our main kitchen. Surplus food from other days is fed to a state-of-the-art biogas unit. One of our stoves is operated with the biogas generated. (Manager, Hotel 2).

All the three supermarket chains interviewed stated that the packed food products which are close to expire are returned to the main stores in the area. One of the major supermarket chains revealed that they are using surplus fruits and vegetables for value-added processing, such as in preparation of salads, juices, and sliced fruits in plastic clamshell containers.

We always keep fresh fruits and vegetables on our shelves for customers. We prepare semi processed salads, packs of sliced fruits and fruit juices at our own food court as convenience products and we use any surplus fruits and vegetables on the shelves, which are good and still edible for preparing these products. This strategy reduces more than half of the fresh produce end in waste bins each day. (Store manager, Supermarket 1).

The two fast-food chains interviewed stated that there is not much excess food collected in their outlets as they freshly prepare meals for customer orders. It is evident these fast-food chains receive orders either directly from customers who visit the outlet or from online orders and freshly prepare meals and food items to serve or deliver. At the end of the day, if there are any surplus food ingredients, they will be discarded to general waste bins to be collected by the municipal council.

### Surplus Food Redistribution

#### The Robin Hood Army

With the aim of addressing two coexisting social problems: food waste and hunger, the Sri Lankan branch of the Robin Hood Army (RHA) started its surplus food distribution service in Colombo in 2016. It is a volunteer organisation that works to collect surplus food from restaurants and community to serve food insecure people. Their volunteers are called “Robins” and mostly comprised of university students and young working professionals. At the time of the interview, the RHA had over 100 registered voluntary members and about 30 out of them were active volunteers.

The RHA recruit volunteers via a sign-up process available in their official website and social media pages. Also, the prospective volunteers must undergo an induction training prior to commencing their services. The induction gives potential volunteers the chance to meet the team and learn logistics of the process. The below quote depicts the relative ease of signing up as a volunteer.

Becoming a Robin is very easy. No experience is needed to join the team. Anyone can simply sign up via this link provided in the RHA website or their social media pages. I signed up in their FB page. (Robin 1, RHA).

The core principles of RHA are (i) not accepting monetary donations, (ii) apolitical, and (iii) being neutral towards religious beliefs. At the time of the interview, their key suppliers were bakeries and cafes in Colombo.

We only accept food donations. Our Robins work to get surplus food from local restaurants and cafes and also from the community to serve less fortunate people. There is a team of our volunteers who visit the potential places of surplus food and to inquire whether they are willing to donate their surplus food. (Lead Robin 2, RHA).

Whenever there is excess food, the donors communicate the location and quantity to the team leaders, who are responsible to share the details with all the volunteers. Surplus food from these donors is collected in the night, before 8.30 pm by volunteers, who coordinate their activities via the social media platform, “WhatsApp.”

We have a WhatsApp group to communicate among our Robins. Once a new robin joins the army and has completed the induction, his or her contact number is added to the group (Lead Robin 1, RHA).

When the donors inform the RHA about the location and quantity of the surplus food available, the Robins share the details on *WhatsApp* group. The available volunteers communicate with each other in the group, go to the places, pick the food, and distribute to the needy people. In this sporadic redistribution practice, the communication frequency using the *WhatsApp* group is unpredictable. The quality of food is checked by the volunteers themselves before they are packed and distributed.

We do sensory evaluations to check the edibility and quality of food we collect. One of the Robins who volunteer to collect food from the donor, smell and taste them in random prior to sharing. If the food is unpacked, we pack them in paper bags which are degradable. (Robin 2, RHA).

The volunteers use their own vehicles in collecting food from donors and in redistributing them among needy people. The sections of society they serve include homeless people, families in need, orphanages, old age homes, and patients from public hospitals. However, donations to patients in hospitals involve fresh food distribution which is out of the scope of the present study and hence excluded from Fig. 3. The surplus food supply and demand in Colombo are unpredictable, which is a characteristic of sporadic redistribution. Before the COVID-19 pandemic, RHA had provided food to roughly 800 meals on a monthly average. At the time of the interview, RHA served around 300–400 meals a month.

#### The Soup Bowl

Another social initiative is “The Soup Bowl,” a front-line organisation, supported by community monetary donations, focuses on feeding needy elders, children, the homeless, and the underprivileged [55]. It is a privately owned charity, which receives donations from well-wishers, family, and friends. The relatively small-scale operation is supported by a group of eight volunteers, at the time of interview. It was started in 2015 with providing lunch packages for the homeless.

In the beginning I used to buy parcels of food and distribute to people begging in the street where I lived. A lot of my friends and colleagues contributed too and I was able to provide meals for several poor. (Founder, The Soup Bowl).

After receiving external funding and support, the organisation was able to expand its operations. It introduced an initiative called “Drop-in Centre” which provides weekly free lunch for people who gather at a community centre every Friday. The beneficiaries of this project include the street cleaners, office cleaners, the retired elders, and street people. Prior to the COVID-19 pandemic in late 2019, this initiative was capable in providing the lunch for more than 125 needy people in a day.

*WeGiveStuffAway* (WGSA) is the surplus food redistribution project of The Soup Bowl charity which was initiated with the motive of reducing food waste through food rescue. They have tied up with grocery stores and local shops and rescue their surplus food that run out of shelf life but are still usable (fruits, vegetables, cereals, canned food etc.). They select the beneficiaries, especially families in need through nominations from public, where anyone can contact them via email or through FB page and suggest the nominees.

We rescue surplus fruits and vegetables, dry rations, canned items and deliver them to economically challenged families. We always invite the general public to nominate people or families who can’t make ends meet and we assure any detail they share to remain confidential. (Founder, The Soup Bowl).

In weekdays, during the night, the WGSA collects surplus vegetables and fruits from supermarkets and unsold food from bakers and distribute them among people in need. The surplus food demand is unpredictable, as the selection of beneficiaries depends on the public nominations.

### Institutional Structure of Colombo’s Food Recue System

Figure 3 shows the institutional structure of the food rescue system in Colombo. The findings of the preceding section suggest that there are missing institutions and links in the flow of surplus food redistribution and the surplus food redistribution activities in Colombo. In particular, the operations are not supported by facilitator organisations and back-line organisations (Fig. 3).

### Motives of Surplus Food Donation and Redistribution

The interviewees were asked to describe and rank their motivations for involving in surplus food donation and redistribution. Majority of the donor interviewees (86%) who represent restaurants and cafes have identified “concern for people” as the main motive for surplus food donation and “concern for the environment” was identified as their second largest motivator. Both hotel managers and two out of three store managers in supermarkets have ranked “concern for the environment” as the main motivation to donate surplus food. Although all surplus food donors interviewed were for-profit organisations, none of them have ranked either “saving money” or “public reputation” in the first place. However, this could be due to social desirability bias prevalent in face-to-face interviews. The majority of the interviewees (66%) who represent front-line organisations have identified “concern for people” as the main motivation for surplus food rescue and “concern for the environment” was identified as their second-ranked motivating factor.

### Food Rescue and Redistribution Challenges

Both front-line organisations outlined in “Surplus Food Redistribution” struggled with the lack of infrastructure and logistics such as refrigerated vehicles and storage facilities with temperature controllers, unpredictable food supply and demand, limited availability of volunteers, and time limitations of volunteers. There are very limited collaborations among surplus food distributing organisations and surplus food donors in Colombo, Sri Lanka. The food donors are reluctant to donate surplus food to a third party as they are feared on reputational risks if the food was not reaching its target group while it was fresh and safe. This fact is supported by findings of [23].

Some restaurants and cafes are reluctant to partner with us although they own surplus food and have the motive to share them with needy people, claiming that we do not have proper logistics to do so. They are afraid that if something goes wrong with our process, it may harm their reputation. (Lead Robin 1, RHA).

Volunteers of RHA and WGSA use their own vehicles to pick and deliver goods to various locations in and around Colombo. Therefore, the main obstacle the front-line organisations face is not having facilities to check the food quality and the absence of logistics to store and transport food in adequate temperature in a way to secure their shelf life. These findings are supported by literature which purports that food rescue efforts are hampered by logistical constraints [20, 56]. Limited availability of resources and well-developed infrastructure were identified as a barrier for operations in food banks [27, 57]. The findings of Reitemeier et al. [23] also support the lack of logistic support for food rescue in Sri Lanka, which is a common problem in developing country food rescue and redistribution operations. Moreover, food rescue schemes in India are constrained by malpractices and inefficiencies hampering the goal of hunger alleviation which includes inadequate service planning, poor resource utilisation, poor budgeting, and corruption [58, 59].

Another key challenge mentioned was the short gap between time of food release from the donors and time of food demand. Before the food is considered waste, the two front-line organisations must collect, sort it out, and deliver to the beneficiaries. Within the limited shelf life, rescued food is directly transported to nominated homes and is typically not used for later weekly meal preparations. This is supported by Nair et al. [20] which states that food rescue organisations collect food from different food providers and deliver them on the same day, instead of storing them, due to the perishability of food products collected.

The lack of human resources is a significant threat to effective food rescue operations [27, 35, 57]. The food rescue operations of the two front-line organisations are challenged by insufficient volunteers participating in food redistribution activities.

Although there are more than 100 Robins registered with RHA Sri Lanka, less than 30 robins are actively involved in distributing surplus food from local shops and cafes to people starving in Colombo. (Lead Robin 1, RHA).

The travel restrictions imposed due to COVID-19 pandemic explains the limited number of volunteers involved in food redistribution. There may be some other factors influencing this situation. The non-profit food rescue organisations operate in the social interest, not cost driven and therefore they do not offer any financial benefits for their volunteers [20]. In most of the developing countries like Sri Lanka, volunteers are motivated by the hope that volunteering creates possibilities in future employment [60]. Whenever the efforts in volunteering are not valued by the job market, people may get less motivated and refrain from volunteering.

Structured and reliable processes for organising frequent collection and distribution trips between donors and beneficiaries are required for effective food redistribution [56]. However, the limited time availability challenged by the short shelf life of donated food and the limited participation of volunteers limit the frequency of collection and distribution trips and therefore, reduce the efficiency in food redistribution activities [27, 57]. An initiative in India have developed a smartphone App to connect with surplus food donors, so that there will be no delays in being notified on the availability of surplus food [42].

Personal preferences and social norms may challenge and perhaps explain the slow expansion of food redistribution operations in Sri Lanka. Food partially consumed by others is perceived as unclean, and it creates a feeling of disgust [61]. Consumers’ personal preferences and feelings influenced by social norms lead to avoiding leftovers in meals despite their economic situations [61]. These social norms explain the uncertainty in demand from beneficiaries namely, families in need, elders’ homes, and orphanages while the majority of people benefited from food redistribution in Colombo are homeless people. This may be a reason for not including food rescue operations in Sri Lanka’s policy statements and goals, as a strategy to manage food wastage or to elevate food security.

Although not directly revealed in interviews, there are certain prejudices against eating leftover food which may hinder surplus food donations and volunteering in food redistribution activities in Sri Lanka. Community attitudes, perceptions, beliefs, and social norms on donating and consuming “used or second-hand food” [62] may influence the food rescue operations. Consumers tend to avoid reusing leftovers due to perceived quality and freshness losses [63–65]. Health-conscious people perceive leftover food as lacking nutrition and could be harmful to health, so therefore, they tend to dispose of surplus food [66, 67] as they consider being healthier is more important than preventing food waste [68].

Due to the limited amount of food donations, unpredictable food supply and demand, and absence of minimum quality and safety standards required for surplus food redistribution, the food rescue organisations in Colombo appear to distribute any food they collect, regardless of its nutritional value. This situation is supported by previous studies which highlight that when the availability of donors is restrained, the food banks end up delivering unsold food from any donor, without considering the nutritional requirements of people in need [69]. Moreover, unpredictability, either on the surplus food supply [70], or on the demand for food from beneficiaries [71] create difficulties with distributing food that meet the nutritional requirements of clients. This is identified as a limitation for a healthy diet [22] and therefore, make it difficult for policy makers in developing action plans to promote food rescue as a solution for food security.

## Conclusions and Policy Implications

The study examined the existing food rescue system in Colombo, Sri Lanka, by conducting in-depth interviews with twenty surplus food donors and redistributors with the aims of providing useful insights on food redistribution activities and developing policies to reduce food waste and to ensure food security in Sri Lanka. The humanitarian concern was the main motive for both surplus food donation and surplus food redistribution, while the second highest motive was the concern for the environment. In addition to these motivations, businesses may see their donation as form of corporate social responsibility, or as a marketing strategy to be viewed favourably by the public [31].

The restaurants and cafes share surplus food among employees and donate surplus food to homeless directly or through limited number of front-line food redistribution organisations. However, the hotels and fast-food chains do not practice above strategies due to reputational risks. The hotels send majority of their surplus food for livestock feed, mainly to piggeries, and use a portion of surplus food in renewable energy generation, while supermarket chains use their surplus fresh produce in value added processing and donate surplus fruits, vegetables, and food ingredients to a local charity, which is a front-line food redistribution organisation.

The findings reveal that the surplus food rescue system in Colombo, Sri Lanka, is not supported by facilitator organisations and back-line organisations. Lacking these facilities increase the risk of food getting spoiled and may increase wastage of rescued food. In the absence of these facilitator organisations, there are gaps in coordination between food donors and front-line organisations. Due to poor coordination between parties, recovered food will not be utilised effectively resulting high food wastage. Therefore, the missing facilitator institutions in food rescue system in Colombo may result in inefficiencies in food rescue operations. Food donors are reluctant to donate surplus food to a third party who experience such challenges as they are feared on reputational risks. To overcome this issue, the organisations could use web platforms and smartphone apps to effectively match food donors with front-line organisations.

Another implication arising from this study is that the need to establish food banks as back-line organisations supporting front-line organisations to provide required food logistics such as cold storage facilities for perishable food redistribution. Moreover, to facilitate effective food redistribution, it is essential to develop structured and reliable processes for frequent collection and distribution trips between donors and beneficiaries. The government authorities responsible in food logistics development such as Ministry of Agriculture and Ministry of Urban Development can partner with the main supermarket chains to establish food banks, with state-of-the-art food storage and transport facilities. The island wide coverage of main supermarket chains will facilitate in expanding the food rescue operations beyond Colombo district and will allow collecting food donations frequently, contributing to the wider adoption of a circular economy approach in the country.

The study revealed that the food rescue system characterises a sporadic redistribution based on the analysis of two front-line organisations of food redistribution currently operating in Colombo. Having a formal agreement between food donors and food rescue organisations is pivotal for an effective and sustainable food redistribution. None of the food donors in Colombo have formal agreements with the food redistribution organisations, as they are not fully satisfied with the logistics in redistributing food and as they are not aware of safety and quality standards maintained by the food redistributors. Therefore, there is no agreed schedule for food donations and pickup which is a challenge for the continuous flow of food rescue operations. An important policy implication arising from this finding is that policy makers should facilitate establishing formal food rescue agreements between large supermarket chains and food redistribution organisations, to convert the redistribution process ongoing. The agreements can include responsibilities and liabilities of both the parties, specific dates that should be considered (e.g. donated before the use-by date), a consent note to donate free of charge, collection schedule, and a list of non-accepted products. The ties between the two parties will be strong in an ongoing redistribution as they have a commitment to continue the relationship [43]. Meantime, the respective government authorities such as Ministry of Health, Ministry of Local Government and Sri Lanka Standards Institution should formulate health and safety guidelines on hygiene requirements, food safety parameters, and minimum quality standards required for surplus food redistribution. Moreover, a transparent monitoring mechanism should also be established to check whether the different parties involve in food redistribution are adhering to these guidelines.

Awareness creation programmes can be organised by the respective government authorities such as the Ministry of Health, Ministry of Local Government, Central Environment Authority, Urban Councils and *Pradheshiya Sabhas* to increase the awareness among food retailers, suppliers, and the general public on existing food redistribution activities and on food safety and quality parameters in donating surplus food in line with formulated health and safety guidelines. This will build trust among food donors in food redistribution activities which can lead to the creation of food banks and will increase the number of volunteers join with the not-for-profit food redistribution organisations. Strengthening food rescue systems in developing countries can make a significant contribution in reducing food insecurity in marginalised communities.

Restrictions imposed due to COVID-19 pandemic limited conducting face-to-face interviews with surplus food redistributors, and therefore, only two organisations participated through phone interviews. Also, the study interviewed only 14 people representing 13 surplus food donors and these people were reluctant to disclose the quantities of surplus food donated. This has limited the study to determine the strength of the relationship between donors and food redistributors quantitatively. More research is needed before generalising the identified framework of flow of surplus food redistribution to other developing countries as the attention to food waste varies considerably according to the domestic circumstances of the country.

## Data Availability

The data that support the findings of this study are available from the authors, upon request.

## Appendix

Table 1

**Table 1:** Description of the sample

	Organisations	Interviewees
Surplus food redistributors	The Soup Bowl	(no. 1) Founder (no. 2) Volunteer
	The Robin Hood Army	(no. 3) Lead volunteer 1 (no. 4) Lead volunteer 2 (no. 5) Volunteer 1 (no. 6) Volunteer 2
Surplus food donors	2 Hotels	(no. 7) Manager, hotel 1 (no. 8) Manager, hotel 2
	2 Restaurants	(no. 9) Manager, restaurant 1 (no. 10) Manager, restaurant 2 (no. 11) Waiter, restaurant 2
	4 Cafes	(no. 12) Owner, café 1 (no. 13) Waiter, café 2 (no. 14) Manager, café 3 (no. 15) Manager, café 4
	3 Supermarket chains	(no. 16) Store manager, supermarket chain 1 (no. 17) Store manager, supermarket chain 2 (no. 18) Store manager, supermarket chain 3
	2 Fast food chains	(no. 19) Staff member, fast food chain 1 (no. 20) Staff member, fast food chain 2

## Footnotes

1 Food insecurity is regarded as a household-level economic and social condition of limited or uncertain access to adequate food (USDA, 2022).

2 Food banks are non-profit organisations which serve as centres for food donations [72] and distribute food through other non-profit institutions which feed food insecure people [73].

## References

[1] Godenau D, Caceres-Hernandez JJ, Martin-Rodriguez G, Gonzalez-Gomez JI (2020). A consumption-oriented approach to measuring regional food self-sufficiency. Food Secur.

[2] FAO, IFAD, UNICEF, WFP, WHO (2021) The State of Food Security and Nutrition in the World 2021. Transforming food systems for food security, improved nutrition and affordable healthy diets for all. Food and Agriculture Organisation, Rome, Italy. https://www.fao.org/documents/card/en/c/cb4474en. Accessed 2 Mar 2022

[3] United Nations Environment Programme (2021) Food and food waste. Sustainable food systems. United Nations Environment Programme. https://www.unep.org/explore-topics/resource-efficiency/what-we-do/sustainable-lifestyles/food-and-food-waste#:~:text=UNEP%20Sustainable%20Food%20Systems%20Programme&text=UNEP%20is%20committed%20to%20accelerating,local%2C%20regional%20and%20international%20level. Accessed 12 May 2022

[4] United Nations (2020) Take action for the sustainable development goals. United Nations. https://www.un.org/sustainabledevelopment/sustainable-development-goals/. Accessed 20 May 2022

[5] Food and Agriculture Organisation (2011) Global food losses and food waste — extent, causes and prevention. Food and Agriculture Organisation, Rome, Italy. https://www.fao.org/3/i2697e/i2697e.pdf. Accessed 5 Mar 2022

[6] Food and Agriculture Organisation (2019) The State of Food and Agriculture 2019. Moving forward on food loss and waste reduction. Food and Agriculture Organisation, Rome, Italy. https://www.fao.org/3/ca6030en/ca6030en.pdf. Accessed 5 Mar 2022

[7] Muswema A, Ramukhwatho F, Oelofse S (2018). Household food waste disposal in South Africa: a case study of Johannesburg and Ekurhuleni. South Afr J Sci.

[8] Roodhuyzen DMA, Luning P, Fogliano V, Steenbekkers LPA (2017) Putting together the puzzle of consumer food waste: towards an integral perspective. Trends Food Sci Technol 68:37–50. 10.1016/j.tifs.2017.07.009

[9] KPMG, Fight Food Waste Cooperative Research Centre (2020) Fighting food waste using the circular economy. KPMG. https://assets.kpmg.com/content/dam/kpmg/au/pdf/2019/fighting-food-waste-using-the-circular-economy-report.pdf. Accessed 20 June 2022

[10] Borrello M, Caracciolo F, Lombardi A, Pascucci S, Cembalo L (2017). Consumers’ perspective on circular economy strategy for reducing food waste. Sustainability (Basel, Switzerland).

[11] Dong L, Liu Z, Bian Y (2021). Match circular economy and urban sustainability: re-investigating circular economy under sustainable development goals (SDGs). Circ Econ Sustain.

[12] Ciccullo F, Cagliano R, Bartezzaghi G, Perego A (2021). Implementing the circular economy paradigm in the agri-food supply chain: the role of food waste prevention technologies. Resour, Conserv Recycl.

[13] Murray A, Skene K, Haynes K (2017). The circular economy: an interdisciplinary exploration of the concept and application in a global context. J Bus Ethics.

[14] Sarti S, Corsini F, Gusmerotti NM, Frey M (2017) Food sharing: making sense between new business models and responsible social initiatives for food waste prevention. Econ Pol Energy Environ 2017(1-2):123–134. 10.3280/EFE2017-001007

[15] Nolin DA (2012). Food-sharing networks in Lamalera, Indonesia: status, sharing, and signaling. Evol Hum Behav.

[16] Garrone P, Melacini M, Perego A, Sert S (2016). Reducing food waste in food manufacturing companies. J Clean Prod.

[17] Arcuri S (2019). Food poverty, food waste and the consensus frame on charitable food redistribution in Italy. Agric Hum Values.

[18] Hecht AA, Neff RA (2019). Food rescue intervention evaluations: a systematic review. Sustainability (Basel, Switzerland).

[19] Vittuari M, De Menna F, Gaiani S, Falasconi L, Politano A, Dietershagen J, Segre A (2017). The second life of food: an assessment of the social impact of food redistribution activities in Emilia Romagna, Italy. Sustainability (Basel, Switzerland).

[20] Nair DJ, Rey D, Dixit VV (2017). Fair allocation and cost-effective routing models for food rescue and redistribution. IISE Transactions.

[21] Canali M, Amani P, Aramyan L, Gheoldus M, Moates G, Östergren K, Silvennoinen K, Waldron K, Vittuari M (2017) Food waste drivers in Europe, from identification to possible interventions. Sustainability 9(1):37. 10.3390/su9010037

[22] Bierwagen MY, GonÇAlves Dias SLF (2021) Food rescue and donation in socioenvironmental policies on tackling food loss and waste: a systematic review. Future Food: J Food, Agric Soc 9(5):27–38. https://www.thefutureoffoodjournal.com/index.php/FOFJ/article/view/409. Accessed 30 May 2022

[23] Reitemeier M, Aheeyar M, Drechsel P (2021) Perceptions of food waste reduction in Sri Lanka's Commercial Capital, Colombo. Sustainability 13(2):838. 10.3390/su13020838

[24] Kogyo Co K (2016) Data collection survey on solid waste management in democratic socialist republic of Sri Lanka. Final Report. Japan International Cooperation Agency. https://openjicareport.jica.go.jp/pdf/12250213.pdf. Accessed 12 Mar 2022

[25] Jayathilake N, Aheeyar M, Drechsel P (2022) Food waste to livestock feed: prospects and challenges for swine farming in peri-urban Sri Lanka. Circ Econ Sustain 2:1301–1315. 10.1007/s43615-022-00168-8 10.1007/s43615-022-00168-8 PMC9002037 35434720

[26] Food and Agriculture Organisation (2018) Assessing and planning city region food system Colombo (Sri Lanka) synthesis report. FAO. https://www.fao.org/3/CA1159EN/ca1159en.pdf. Accessed 20 Mar 2022

[27] Dubey N, Tanksale A (2022). A study of barriers for adoption and growth of food banks in India using hybrid DEMATEL and Analytic Network Process. Socio-econ Plan Sci.

[28] Halog A, Anieke S (2021). A review of circular economy studies in developed countries and its potential adoption in developing countries. Circ Econ Sustain.

[29] Aiello G, Enea M, Muriana C (2015). Alternatives to the traditional waste management: food recovery for human non-profit organizations. Int J Oper Quant Manag.

[30] González-Torre PL, Coque J (2016). From food waste to donations: the case of marketplaces in northern Spain. Sustainability.

[31] Vlaholias E, Thompson K, Every D, Dawson D (2015). Charity starts … at work? Conceptual foundations for research with businesses that donate to food redistribution organisations. Sustainability (Basel, Switzerland).

[32] Albizzati PF, Tonini D, Chammard CB, Astrup TF (2019). Valorisation of surplus food in the French retail sector: environmental and economic impacts. Waste Manage.

[33] Reynolds CJ, Piantadosi J, Boland J (2015). Rescuing food from the organics waste stream to feed the food insecure: an economic and environmental assessment of Australian food rescue operations using environmentally extended waste input-output analysis. Sustainability.

[34] Alexander C, Smaje C (2008). Surplus retail food redistribution: an analysis of a third sector model. Resour Conserv Recycl.

[35] Mousa TY, Freeland-Graves JH (2017). Organizations of food redistribution and rescue. Public Health.

[36] Sert S, Garrone P, Melacini M, Perego A (2018). Corporate food donations: altruism, strategy or cost saving?. Br Food J.

[37] Damiani M, Pastorello T, Carlesso A, Tesser S, Semenzin E (2021). Quantifying environmental implications of surplus food redistribution to reduce food waste. J Clean Prod.

[38] Bergström P, Malefors C, Strid I, Hanssen OJ, Eriksson M (2020). Sustainability assessment of food redistribution initiatives in Sweden. Resources (Basel).

[39] Harvey J, Smith A, Goulding J, Illodo IB (2020). Food sharing, redistribution, and waste reduction via mobile applications: a social network analysis. Ind Mark Manage.

[40] European Commission (2017) EU guidelines on food donation*. *European Union. https://circulareconomy.europa.eu/platform/en/toolkits-guidelines/eu-guidelines-food-donation. Accessed 27 May 2022

[41] Sundgren C (2020). Supply chain structures for distributing surplus food. Int J Logist Manag.

[42] Agrawal VS, Nag A (2013). Sustainable food waste prevention strategies to achieve food security in India. Int J Agric Food Sci Technol.

[43] Sundgren C (2022) Circular supply chain relationships for food redistribution. J Clean Prod 336:130393. 10.1016/j.jclepro.2022.130393

[44] Colombo Municipal Council (2015) City Profile. Colombo Municipal Council. https://www.colombo.mc.gov.lk/colombo.php. Accessed 30 Mar 2022

[45] Gunawardhana N, Ginigaddara G (2021). Household food security of urban slum dwellers: a case study in Colombo municipality, Sri Lanka. J Food Chem Nanotechnol.

[46] Gunetilleke N, Cader AA, Fernando M (2004) Understanding the dimensions and dynamics of poverty in underserved settlements in Colombo: The Centre for Poverty Analysis, Sri Lanka. https://www.cepa.lk/wp-content/uploads/2004/10/Understanding-the-Dimensions-03-min-min.pdf. Accessed 26 May 2022

[47] Deyshappriya R (2019). An empirical analysis on food insecurity in Sri Lanka. Empir Econ Rev.

[48] Aheeyar M, Reitemeier M, Bucatariu C, Bandara A, Thiel F, Jayathilake N, and Drechsel P (2020) Food waste in Sri Lanka: an analysis of the applicable urban regulatory framework*.* Second Draft. https://docplayer.net/206442031-Food-waste-in-sri-lanka-an-analysis-of-the-applicable-urban-regulatory-framework.html. Accessed 15 March 2022

[49] Esham M, Garforth C (2013). Agricultural adaptation to climate change: insights from a farming community in Sri Lanka. Mitig Adapt Strat Glob Change.

[50] Mayadunne G, Romeshun K (2013). Estimation of prevalence of food insecurity in Sri Lanka. Sri Lankan J Appl Stat.

[51] World Food Program (2022). Sri Lanka — country brief.

[52] Global Hunger Index (2021) Sri Lanka — global hunger index 2021. Global Hunger Index. https://www.globalhungerindex.org/pdf/en/2021/Sri-Lanka.pdf. Accessed 17 Jan 2022

[53] Fernando S, Santini G (2018) Food security and nutrition in city region food system planning, Colombo, Sri Lanka, policy brief. Food Agric Organ, Rome, Italy. https://ruaf.org/assets/2019/12/Policy-brief-on-food-security-Colombo.pdf. Accessed 3 Mar 2022

[54] World Bank (2022) Reshaping norms: a new way forward. South Asia Economic Focus: Spring 2022. World Bank Group, Washington, USA. https://openknowledge.worldbank.org/bitstream/handle/10986/37121/9781464818578.pdf?sequence=13&isAllowed=y. Accessed 4 June 2022

[55] Jayawardena T (2019) The Soup Bowl: more than just a wholesome meal. https://www.sundaytimes.lk/190922/plus/the-soup-bowl-more-than-just-a-wholesome-meal-369114.html. Accessed 21 Jan 2021

[56] Garrone P, Melacini M, Perego A (2014). Surplus food recovery and donation in Italy: the upstream process. Br Food J.

[57] Gharehyakheh A, Sadeghiamirshahidi N (2018) A sustainable approach in food bank logistics. Proc Int Annu Conf Am Soc Eng Manag. https://www.researchgate.net/publication/328677026_A_Sustainable_Approach_in_Food_Bank_Logistics. Accessed 2 June 2022

[58] Chakraborty S, Sarmah S (2019). India 2025: the public distribution system and national food security act 2013. Dev Pract.

[59] Khera R (2006). Mid-day meals in primary schools: achievements and challenges. Econ Pol Wkly.

[60] Walt G, Perera M, Heggenhougen K (1989). Are large-scale volunteer community health worker programmes feasible? The case of Sri Lanka. Soc Sci Med.

[61] Aleshaiwi A, Harries T (2021). A step in the journey to food waste: how and why mealtime surpluses become unwanted. Appetite.

[62] Andrews L, Kerr G, Pearson D, Mirosa M (2018). The attributes of leftovers and higher-order personal values. Br Food J.

[63] Cappellini B (2009). The sacrifice of re-use: the travels of leftovers and family relations. J Consum Behav: Int Res Rev.

[64] Porpino G, Parente J, Wansink B (2015). Food waste paradox: antecedents of food disposal in low income households. Int J Consum Stud.

[65] Schanes K, Dobernig K, Gözet B (2018). Food waste matters—a systematic review of household food waste practices and their policy implications. J Clean Prod.

[66] Savelli E, Francioni B, Curina I (2019). Healthy lifestyle and food waste behavior. J Consum Mark.

[67] van Geffen L, van Herpen E, Sijtsema S, van Trijp H (2020). Food waste as the consequence of competing motivations, lack of opportunities, and insufficient abilities. Resour, Conserv Recycl: X.

[68] Watson M, Meah A (2012). Food, waste and safety: negotiating conflicting social anxieties into the practices of domestic provisioning. Sociol Rev.

[69] Tarasuk V, Eakin JM (2005). Food assistance through “surplus” food: Insights from an ethnographic study of food bank work. Agric Hum Values.

[70] Bazerghi C, McKay FH, Dunn M (2016). The role of food banks in addressing food insecurity: a systematic review. J Community Health.

[71] Lee D, Sönmez E, Gómez MI, Fan X (2017). Combining two wrongs to make two rights: mitigating food insecurity and food waste through gleaning operations. Food Pol.

[72] Handforth B, Hennink M, Schwartz MB (2013). A qualitative study of nutrition-based initiatives at selected food banks in the feeding America network. J Acad Nutr Diet.

[73] Martins I, Guedes T, Rama P, Ramos J, Tchemisova T (2011). Modelling the problem of food distribution by the Portuguese food banks. Int J Math Model Numer Optimisation.

